# Three-Dimensional Structural Aspects of Protein–Polysaccharide Interactions

**DOI:** 10.3390/ijms15033768

**Published:** 2014-03-03

**Authors:** Masamichi Nagae, Yoshiki Yamaguchi

**Affiliations:** Structural Glycobiology Team, Systems Glycobiology Research Group, RIKEN-Max Planck Joint Research Center, RIKEN Global Research Cluster, 2-1 Hirosawa, Wako, Saitama 351-0198, Japan; E-Mail: mnagae@riken.jp

**Keywords:** polysaccharide, lectin, 3D structure, polylactosamine, galectin, carbohydrate binding module, β-glucan, polysialic acid, antibody, affinity, hyaluronan, CD44

## Abstract

Linear polysaccharides are typically composed of repeating mono- or disaccharide units and are ubiquitous among living organisms. Polysaccharide diversity arises from chain-length variation, branching, and additional modifications. Structural diversity is associated with various physiological functions, which are often regulated by cognate polysaccharide-binding proteins. Proteins that interact with linear polysaccharides have been identified or developed, such as galectins and polysaccharide-specific antibodies, respectively. Currently, data is accumulating on the three-dimensional structure of polysaccharide-binding proteins. These proteins are classified into two types: exo-type and endo-type. The former group specifically interacts with the terminal units of polysaccharides, whereas the latter with internal units. In this review, we describe the structural aspects of exo-type and endo-type protein-polysaccharide interactions. Further, we discuss the structural basis for affinity and specificity enhancement in the face of inherently weak binding interactions.

## 1. Introduction

**Figure 1.**
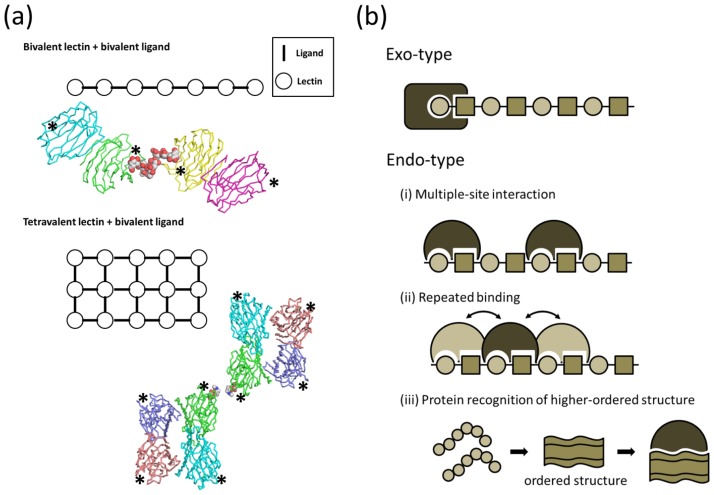
(**a**) Schematic representations of bivalent lectin-bivalent ligand (type I) and tetravalent lectin-bivalent ligand (type II) complexes. Crystal packings are shown for bivalent galectin-1 dimer in complex with biantennary complex-type glycan (type I, upper panel, PDB code 1SLB), and tetravalent soybean agglutinin tetramer in complex with binantennary complex-type glycan (type II, lower panel, PDB code 1SBE). Protein and carbohydrate molecules are shown in wire and sphere models, respectively, and arbohydrate binding sites are shown with asterisks; (**b**) Schematic representations of exo-type (upper) and endo-type (lower) interactions. The affinity enhancement strategies of endo-type lectins: (i) multiple-site interaction; (ii) repeated binding; and (iii) protein recognition of ordered/higher-ordered polysaccharide structures.

Compared with protein–protein interactions, the binding between proteins and individual, simple carbohydrate ligands is weak. Carbohydrate-protein interaction often requires affinity enhancement in order to attain biological significance. Binding affinity can be substantially increased by multivalent binding of carbohydrate ligand and/or carbohydrate-binding protein through the cluster effect [1]. Here, multivalent carbohydrate chains and clustered carbohydrate-binding proteins form lectin-carbohydrate lattices. Examples include bovine galectin-1 and soybean agglutinin (SBA) [2]. Dimeric bovine galectin-1 complexed with biantennary complex-type glycan displays multiple cross-links through its branched glycan termini [3]. SBA is a tetrameric legume lectin that uniquely complexes with multiantennary carbohydrates [4,5] (Figure 1a). The biological importance of the galectin/lectin lattice has been reviewed [6].

The mechanism of affinity enhancement among linear repeating polysaccharides, such as polylactosamine, hyaluronan, β-glucan, and polysialic acid, which are ubiquitous among living organisms, is quite distinct. Linear polysaccharides serve as a scaffold for crosslinking to polysaccharide-binding proteins, thereby enhancing the affinity of specific binding partners. Lectins that bind to linear polysaccharides are classified as either exo- or endo-types (Figure 1b). Exo-type lectins interact with terminal units of polysaccharides, and bind glycan chains in partially sealed clefts. Lectins of this type specifically bind to the terminal cap structure. In contrast, endo-type lectins interact with internal units of polysaccharides using open clefts. Endo-type lectins enhance binding affinity to polysaccharide ligands by three mechanisms: (1) Multiple-site interactions; (2) Repeated binding; (3) Recognition of ordered/higher-ordered polysaccharide structure.

Tighter binding of endo-type proteins to longer polysaccharides is often achieved by combining all three mechanisms. Multiple-site interaction occurs when a long polysaccharide binds two or more lectins simultaneously. Such interactions can be identified by isothermal titration calorimetry and the stoichiometry of binding analyzed using a Scatchard plot. Repeated binding implies that one lectin repeatedly dissociates and rebinds or slides on the polysaccharide. The apparent affinity is enhanced by slowing the dissociation of the protein. This mechanism can be identified by kinetic analysis, using techniques such as surface plasmon resonance. This mode of affinity enhancement requires that the protein has ligand-binding pockets open at both ends. The third mechanism is called the conformational epitope hypothesis, but remains speculative. In this interaction, a polysaccharide of a certain chain length forms a higher-ordered conformation, such as a helix, that assists in protein recognition and tighter binding. The chain length at which the polysaccharide forms a higher-ordered structure will depend on the glycosidic linkage and sugar type.

The shorter sugar chains, the oligosaccharides, are highly soluble in water as are many of the longer ones, the polysaccharides. However, some polysaccharides are insoluble—these include curdlan (linear β1–3 glucan), cellulose (β1–4 glucan), and chitin/chitosan (β1–4 linked GlcNAc/GlcN polymer). Insoluble polysaccharides such as cellulose and chitin play structural roles in plants, fungi, and insects. Interaction between proteins and cellulose has been studied extensively [7] and reviewed [8,9]. Type-A cellulose-binding modules (CBMs) use a flat surface populated with aromatic residues to bind to crystalline cellulose whereas type-B CBMs use a deep groove to bind individual twisted glucan chains found in disordered cellulose. Human chitin-binding proteins have been reported recently. Human YKL-39 and -40, also designated as cartilage glycoproteins, bind preferentially to chitooligosaccharides [10–12]. The specificity of YKL-40 for chitin is critically dependent on the length of the oligosaccharide. Although we now have 3D structural details of the chitooligosaccharide–protein interaction, the physiological role of these proteins remains poorly understood.

In this review, we highlight four types of soluble oligo/polysaccharides—polylactosamine, hyaluronan, short β1–3 glucans, and α2–8 polysialic acids—and summarize their three-dimensional (3D) structures and modes of protein interaction.

## 2. Atomic Details of the Polylactosamine and Galectin Interaction

Polylactosamine (polyLacNAc) contains repeating *N*-acetyllactosamine (LacNAc) units (3Galβ1–4GlcNAcβ1–)_n_ as glycan extensions of cell-surface glycolipids and glycoproteins. Molecular modeling of low-energy polyLacNAc conformers demonstrates that the β1–4 linkage adopts an extended conformation [13]. Galectins comprise a family of evolutionarily conserved β-galactoside-specific lectins [14] and are binding partners of polyLacNAc. Galectins form multivalent complexes with cell-surface glycoconjugates and in so doing transmit various signals intracellularly to regulate cellular activation, differentiation, and survival [6]. The binding affinity between galectins and polyLacNAc has been comprehensively analyzed by frontal affinity chromatography (FAC) [15]. Galectin-3, the *C*-terminal carbohydrate recognition domain (CRD) of galectin-8, and the *N*- and *C*-terminal CRDs (NCRD and CCRD) of galectin-9 bind longer oligolactosamines tighter, while galectin-1, -2, and -7 and the NCRD of galectin-8 bind with equal strength to any length [15]. Accordingly, the former group is classified as endo-type, while the latter as exo-type. A structural comparison between endo- and exo-type binding highlights the structural differences that define their different interaction modes (see below).

**Figure 2.**
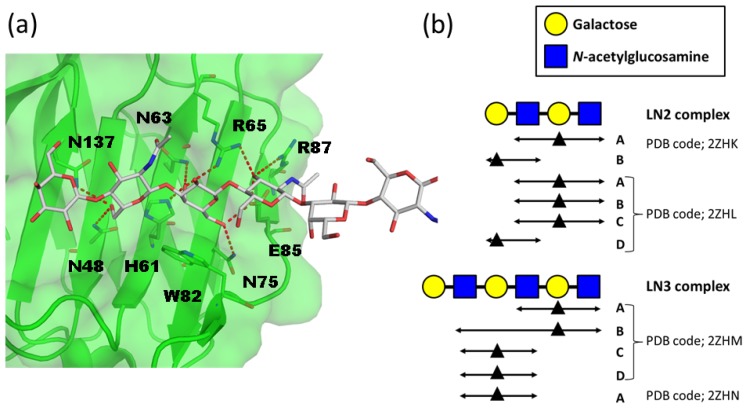
Polylactosamine recognition by galectins. (**a**) Structure of human galectin-9 *N*-terminal carbohydrate recognition domain (NCRD)–LacNAc trimers (LN3) complex (PDB code 2ZHN). The protein molecule is shown in semi-transparent surface and ribbon models. The amino-acid residues that interact with carbohydrate residues are shown in rod models. Hydrogen bonds are indicated by red dotted lines; (**b**) Schematic representation of the interaction modes of LN2 and LN3 complexes. Galactose residues that interact with conserved key residues in galectin-9 NCRD are shown as black triangles. Carbohydrate residues that interact with galectin-9 NCRD are indicated by double head arrows.

Crystal structures of human galectin-9 NCRD complexed with LacNAc dimers (LN2) and LacNAc trimers (LN3) were solved with two different types of crystal packing [16]. The overall structure of galectin-9 NCRD is a β-sandwich fold, and the protein interacts with glycan on its concave surface (Figure 2a). Galectin-9 NCRD interacts with a LacNAc unit in the LN2 and LN3 complexes in two ways (Figure 2b). In the LN2 complex, one molecule (molecule A) interacts with the reducing end of the LacNAc repeat, while the other molecule (molecule B) interacts with the non-reducing end (PDB codes 2ZHK and 2ZHL). In the LN3 complex, one molecule binds to the second LacNAc unit from the non-reducing end, while the other binds a third LacNAc unit (PDB codes 2ZHM and 2ZHN). Thus, human galectin-9 NCRD interacts with three consecutive sugar residues (GlcNAcβ1–3Galβ1– 4GlcNAc) irrespective of their positions within the polyLacNAc chain. Accordingly, it is characterized as an endo-type lectin for polyLacNAc. The dissociation constants of human galectin-9 NCRD for LN2, LN3, and LacNAc pentamers (LN5) are 3.0, 0.81, and 0.12 μM, respectively [15]. The increased affinity for longer chains is explained by both multiple-site interaction and repeated binding.

**Figure 3.**
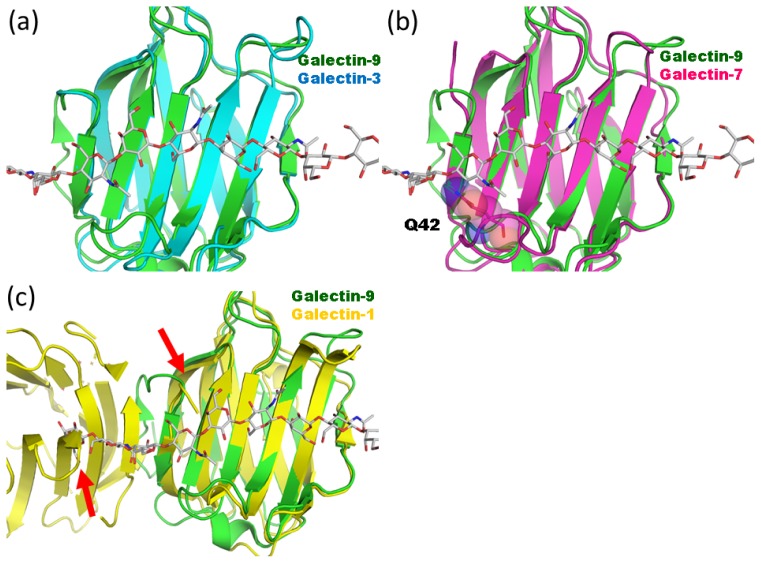
Structural comparison of galectin CRDs. (**a**) Structural superpositions between hypothetical galectin-9 NCRD-LN5 complex (green) and human galectin-3 CRD (cyan; PDB code 1A3K) [17]; (**b**) Structural comparison with human galectin-7 CRD (magenta; PDB code 1BKZ) [18]. The Gln42 side chain, which collides with LN5, is shown in rod model; (**c**) Structural comparison with human dimeric galectin-1 CRD (yellow; PDB code 3OYW) [23]. Possible steric clashes are indicated with red arrows.

A structural comparison between galectin-9 NCRD and other galectins highlights the difference between endo- and exo-type lectins. Human galectin-3 CRD (PDB code 1A3K) [17] can be structurally superimposed onto the human galectin-9 NCRD-LN2 or -LN3 complex without structural conflicts between galectin-3 and the respective oligosaccharides (Figure 3a). This may explain galectin-3’s function as an endo-type lectin for polyLacNAc. In contrast, structural superposition of galectin-7 CRD (PDB code 1BKZ) [18] onto the LN3 complex shows significant steric clash between LN3 and the Gln42 side chain; thus, galectin-7 can only bind to terminal polyLacNAc units (Figure 3b). Molecular dynamics simulations suggest that galectin-8 CCRD interacts with internal LacNAc units of a lactosamine chain [19]. Actually structural superposition of galectin-8 CCRD (PDB code 3VKM) [20] and galectin-9 NCRD LN complexes shows no apparent steric clash.

Galectin-1 primarily interacts with the terminal LacNAc unit of a polyLacNAc chain [21,22]. Galectin-1 CRD monomer superimposes on galectin-9 NCRD-LN2 and -LN3 complexes without steric clash. However, the galectin-1 CRD dimer sterically interferes with the LN chain (Figure 3c). Possibly, formation of a galectin-1 dimer converts it into an exo-type lectin for polyLacNAc, a form that cannot accommodate a longer LN chain. The monomer-dimer equilibrium of galectin-1 in solution may regulate the binding to longer LN chain.

## 3. Structural Studies of CD44 and Hyaluronan (HA) Complex

Hyaluronan is an unbranched polysaccharide comprising repeating disaccharides of GlcNAc and glucoronic acid (GlcUA) connected by β1–3 and β1–4 linkages, (–3GlcNAcβ1–4GlcUAβ1–)_n_. Hyaluronan is localized in the extracellular compartment of most tissues and is a major component of cartilage and synovial fluid. Hyaluronan has diverse biological roles in vertebrates, such as being a vital structural component of connective tissue, the formation of loose hydrated matrices, immune cell adhesion and activation, and a role in intracellular signaling. Hyaluronan is a unique glycosaminoglycan since it is not sulfated. Hyaluronan is likely to be highly dynamic in solution, adopting a large number of low energy states [24].

**Figure 4.**
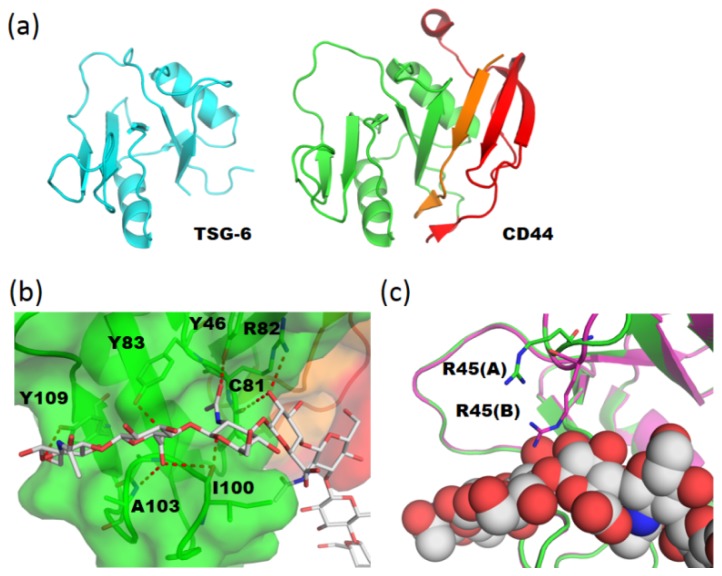
(**a**) Overall structures of tumor necrosis factor stimulating gene-6 (TSG-6) (PDB code 1O7B, [35], left panel) and CD44 (PDB code; 2JCQ, [31], right panel) Link modules. The extended *N*- and *C*-terminal regions of CD44 are colored in orange and red, respectively; (**b**) Close up view of CD44–HA_8_ complex (PDB code 2JCQ). The protein molecule is shown in semi-transparent surface and ribbon models. The amino-acid residues that interact with carbohydrate residues are shown in rod models. Hydrogen bonds are indicated by red dotted lines; (**c**) Structural superposition of two crystal forms of CD44-HA_8_ complex (crystal form A; green, 2JCQ, crystal form B; magenta, 2JCR). HA_8_ molecule is shown in sphere model.

Numerous hyaluronan-binding proteins have been identified [25]. The proteins contain a common structural domain, termed a Link module, which is involved in ligand binding. The Link module, also referred to as a proteoglycan tandem repeat, was first identified in the link protein isolated from cartilage and classified into three subgroups (types A, B, and C) on the basis of the size of their hyaluronan-binding domains [26]. The size of the binding domain appears to correlate broadly with the length of hyaluronan recognized. Structural analyses of HA binding proteins have been limited to tumor necrosis factor stimulating gene-6 (TSG-6) (type A) and CD44 (type B) (Figure 4a).

CD44 is a principal cell surface receptor for hyaluronan and has diverse functions including the attachment, organization, and turnover of extracellular matrix at the cell surface and mediation of the migration of lymphocytes during inflammation [27]. CD44 exists in numerous isoforms due to alternative splicing of 10 variant exons in different combinations. The most abundant standard isoform of CD44 is composed of an extracellular hyaluronan binding domain (HABD), a membrane proximal stalk region, a transmembrane region, and an intracellular *C*-terminal region. The *C*-terminal region is known to interact with cytoskeletal proteins and to link extracellular CD44-HA association with intracellular signaling cascades.

Structural analyses of CD44 HABD have been independently reported by two groups [28–31]. The three-dimensional structures of CD44 HABD in ligand-free state were revealed by X-ray (PDB code 1UUH (human), 2JCP (mouse)) and NMR studies (PDB code 1POZ (human)). CD44 HABD comprises two orthogonally disposed α-helices and ten β-strands organized into a single long, curving β-sheet with a pair of disulfide bridges (Figure 4a). Its fold shows resemblance to tumor necrosis factor stimulating gene-6 (*TSG-6*) Link module but possesses additional *N*- and *C*-terminal extended regions (Figure 4a).

In CD44 HABD-HA_8_ complex (PDB code 2JCQ (type A complex) and 2JCR (type B complex)), seven carbohydrate residues of the octasaccharide could be modeled into the crystal structure—the GlcUA at the reducing end could not be accommodated [31]. The HA chain straddles the cavity of CD44. CD44 interacts strongly with two repeats (tetrasaccharides) at the non-reducing end. These residues are buried in a shallow groove of CD44 and stabilized by a hydrogen-bonded network (Figure 4b). All amino acid residues which make contacts with the carbohydrate are located within the Link module and there is no contribution from other β-strands to ligand binding. There are two crystal forms, termed type A and B complexes. The most significant difference between the two crystal forms is the location of the Arg45 side chain relative to HA_8_ (Figure 4c). In the type A complex structure, there is no contact between Arg45 and HA_8_, while in the type B there is a hydrogen bond between them. It was suggested that type A and type B represent low-affinity and high-affinity states, respectively.

Solution NMR analysis shows that the *C*-terminal segment of HABD has enhanced flexibility in the HA-bound state [30], although the corresponding region of a crystal structure of the HABD-HA_8_ complex adopts an ordered conformation. Moreover, the solution conformation of Arg45 is not uniform even in the presence of HA oligomer. The conformations of the *C*-terminal segment in the unbound and HA-bound states were designated as “ordered” and “partially disordered” conformations, respectively. The transition between “ordered” to “partially disordered” conformations may account for switching from a low to a high affinity form under conditions of shear flow, as required for normal leukocyte trafficking [32]. Further studies will clarify the relationship between these conformation transitions and affinity regulation.

Chain-length-dependent interaction is also reported for CD44. Monovalent binding is observed for chain lengths between HA_6_ and HA_18_, whereas divalent binding occurs in lengths of more than HA_20_ [33]. Interestingly, CD44 shows increased affinity for hyaluronan when the latter is in complex with the TSG-6 link module [34]. It seems protein-protein interactions reinforce protein–carbohydrate interactions of CD44 although the structural basis is still unknown.

## 4. Atomic Characterization of the Interaction between Carbohydrate-Binding Modules and β1–3 Glucan

β1–3 Glucan is a major component of the fungal cell wall and is not synthesized in humans. The innate immune system in humans targets β-glucan as a non-self component. β-glucan assumes a triple-helical structure according to data from X-ray fiber diffraction [36]. In a widely accepted hydrogen-bonding model, one β1–3 glucan chain forms intramolecular hydrogen bonds with the other two chains perpendicular to the axis of the triple helix [37]. Modeling studies of laminarin demonstrated an energetically favorable helical configuration with ϕ and φ angles of −72° and 108°, respectively [38]. Carbohydrate-binding module (CBM) families 4, 6, 39, 43, 52, and 56 interact with β1–3 glucans (CAZy database, http://afmb.cnrs-mrs.fr/CAZY/index.html). To date, 3D structural information on β-glucan binding is limited to CBM4, 6, and 39.

**Figure 5.**
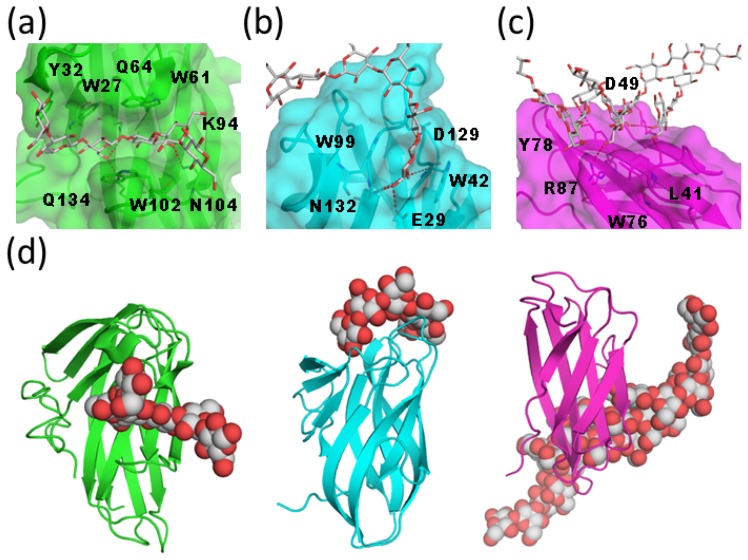
Interaction between cellulose-binding module (CBM) and β1–3 glucan. (**a**) Close-up view of the ligand-binding site of *Tm*CBM4-2 in complex with laminariheptaose (PDB code 1GUI) [39]. Protein molecule is shown in semi-transparent surface and ribbon models. Carbohydrate is shown in rod model; (**b**) Close-up view of the ligand-binding site of *Bh*CBM6 in complex with laminarihexaose (PDB code 1W9W) [40]; (**c**) Close-up view of the ligand-binding site of βGRP-N in complex with laminarihexaose (PDB code 3AQX) [43]; Hydrogen bonds are indicated by red dotted lines; (**d**) Structural comparison of three CBMs, *Tm*CBM4-2 (left), *Bh*CBM6 (middle) and βGRP-N (right). Protein and carbohydrate are shown in ribbon and sphere models, respectively.

CBM4 binds to xylan, amorphous cellulose, β1–3/β1–4 mixed glucan, and β1–3 glucan but not to crystalline cellulose. The crystal structure of CBM4 from *Thermotoga maritima* Lam16A (*Tm*CBM4-2) in complex with laminariheptaose has been reported (PDB code 1GUI) [39]. The *Tm*CBM4-2 structure includes a β-jelly roll with ten β-strands and forms a long, shallow groove (Figure 5a). The carbohydrate-binding site is a U-shaped depression with high-sided walls formed by loops between the β-strands. *Tm*CBM4-2 interacts with glucose residues located in the middle of the glucan chain. Thus, it acts as an endo-type lectin for the β-glucan chain.

CBM6 is found in various enzymes with activities against xylan, mannan, agarose, arabinans, and β-glucans. BH0236 from *Bacillus halodurans* has laminariase activity and possesses CBM6 (*Bh*CBM6) in the *C*-terminal region. The crystal structure of *Bh*CBM6 in complex with laminarihexaose was previously reported (PDB code 1W9W) [40]. The overall structure of *Bh*CBM6 adopts a β-sandwich fold with five- and four-stranded β-sheets (Figure 5b). The binding cleft is occluded at one end, which prevents binding of linear polysaccharides and proper orientation of the sugar at this site, thus conferring specificity for the non-reducing ends of glycans. The terminal glucose residue at the non-reducing end is sandwiched between Trp42 and Trp99 and is recognized via a hydrogen-bond network. In addition, the OH3 group at the non-reducing end interacts with the Asn132 side chain; thus, *Bh*CBM6 interacts with the non-reducing end of the β1–3 glucan chain and acts as an exo-type lectin for the β-glucan chain.

The β1–3 glucan-recognition protein (β-GRP) is classified as a CBM39 and contains an *N*-terminal β-glucan-binding domain and a *C*-terminal β1–3 glucanase-like domain that lacks enzymatic activity [41]. Solution and crystallographic analyses of β-GRP have been performed [42–44]. The crystal structures of the *N*-terminal domain of βGRP (βGRP-N) in complex with laminarihexaose provided evidence for the recognition mechanism (PDB codes 3AQX and 3AQY) [43]. The overall structures of *Plodia interpunctella* and *Bombyx mori* βGRP-N are immunoglobulin (Ig)-like β-sandwich folds composed of three- and five-stranded β-sheets (Figure 5c). One βGRP-N simultaneously interacts with three structured laminarihexaoses via six glucose residues (two from each chain). Since the spatial arrangement of the laminarihexaose bound to βGRP-N is nearly identical to that of a β1–3 glucan triple-helical structure, βGRP-N exhibits high affinity for “triple-helical” β-glucans.

A structural comparison of three CBMs reveals distinct interaction modes despite a common overall fold and binding preference for β1–3 glucans. CBM4 accepts single-chain ligands, while CBM6 interacts with the termini of ligands (Figure 5d). In contrast, CBM39 binds to a conformational epitope formed by triple-helical β-glucans through the flat surface on the convex side. The various interaction modes may reflect the differing physiological roles of CBMs. The conformational-epitope mode of recognition has also been proposed for Dectin-1, which is a C-type lectin-like receptor for longer β1–3 glucans (degree of polymerization (DP) > 10) [45]. Nuclear magnetic resonance (NMR) of medium-sized β-glucan oligomers (DP ≈ 25) suggested the presence of weak hydrogen bonds, which may contribute to the formation of the helix recognized by Dectin-1 [46]. Banana lectin (Banlec) is a dimeric plant lectin from the jacalin-related family and also known to interact with β-glucan. Interestingly, Banlec binds to laminaribiose (Glcβ1–3Glc) mainly through the reducing terminus [47]. A variety of recognition modes of β-glucan by lectins implies this carbohydrate has various physiological roles.

## 5. Interaction between Polysialic Acid and Antibodies

Polysialic acid (polySia) is a linear homopolymer of α2–8- or α2–9-linked sialic acid with a DP of 3 to >400 residues [48,49]. PolySia was discovered as an abundant carbohydrate component in the developing mammalian brain [50] and plays a major role in the development, morphogenesis, and function of various neural systems. A number of antibodies that are specific for oligo/polysialic acid have been developed and are classified into three groups (groups I–III) based on their DP-dependent antigenic specificity [49]. Group I are antibodies that recognize chains of α2–8-linked polySia with a DP ≥ 8. Group II antibodies (termed anti-oligo plus polySia antibodies) recognize di/oligoSia with a DP of two to seven as well as polySia chains. Group III antibodies (termed anti-di/oligoSia antibodies) recognize di- and oligoSia with a DP of 2–4, but not polySia. There is limited structural information on the interactions between oligo/polySia and antibodies. Recently, biochemical and structural analyses of the anti-oligo and polySia antibodies and their fragments 735 (group I), 12E3 (group II), and A2B5 (group III) provided atomic details of antigen recognition [51,52].

**Figure 6.**
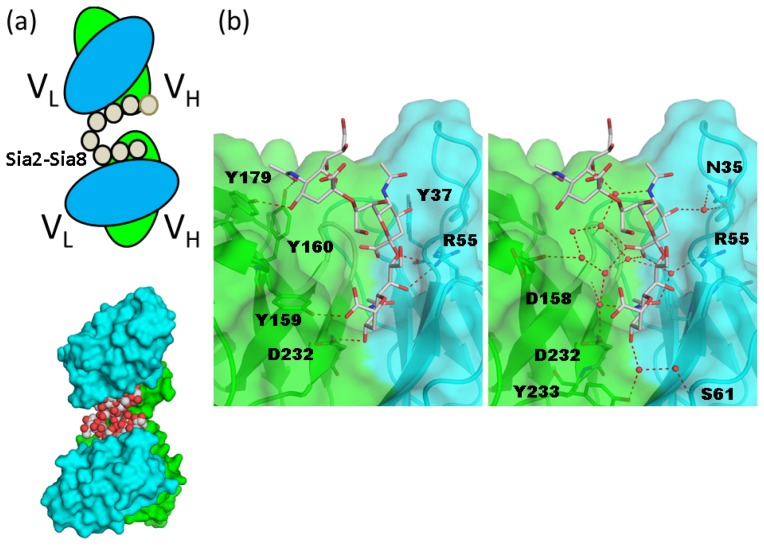
Structural analysis of anti-polysialic acid antibody and oligosialic acid. (**a**) Schematic representation of a single-chain variable fragment of mAb735 (scFv735) in complex with octasialic acid is shown in the upper panel (PDB code 3WBD) [52]. Two proteins and one carbohydrate in the asymmetric unit are shown in the surface and sphere models, respectively (lower panel); (**b**) Close-up view of the carbohydrate-recognition site. Direct and water-mediated interactions between scFv735 and trisialic acid are shown in the left and right panels, respectively. Hydrogen bonds are shown as red dotted lines. Water molecules that bridge scFv735 and trisialic acid are shown as red spheres.

A murine monoclonal antibody mAb735 (group I) has a unique preference for longer polySia polymers (DP > 10). Based on a model of polySia docked in a mAb735 Fab, it was proposed that helical polySia fits into a crevice formed by the CDR loop regions [53]. The crystal structure of the single-chain variable fragment of mAb735 (scFv735) in complex with octasialic acid (PDB code 3WBD) [52] was recently reported. In the asymmetric unit, two scFv735 molecules associate with a single octasialic acid (Figure 6a). In both complexes of the unit, all complementarity-determining regions except for L3 interact with three of eight consecutive sialic acid residues. scFv735 forms hydrogen bonds with oligosialic acid residues by direct and water-mediated interactions (Figure 6b). scFv735 interacts with ligands by an endo-type mechanism, and several scFv735 molecules can bind simultaneously to the long polysialic acid chain. The dissociation constants for scFv735 with a series of oligo/polySia were 1.7 × 10^−3^, 6.5 × 10^−4^, 3.0 × 10^−4^, and 3.5 × 10^−6^ for DP4, DP5, DP6, and polysialic acid (DP = 80–130), respectively. The increase in affinity from DP4 to 6 likely results from the decreased off-rate of the protein. On average, six molecules bind to a single polysialic acid chain; thus, multiple binding contributes to the affinity for polysialic acid. The contribution of helix formation of polysialic acid should be carefully considered, since the interaction between scFv735 and polysialic acid is enthalpically driven (Δ*H*: 80.1 kcal/mol) with an unfavorable entropic contribution (*T*Δ*S*: −72.7 kcal/mol). The unfavorable entropy may result from the loss of conformational flexibility of the polysialic acid. In fact, the dihedral angles of the trisialic acid unit directly interacting with scFv735 are not uniform, indicating that mAb735 does not strictly favor the helical conformation, as once thought.

Saturation transfer difference NMR and transferred nuclear Overhauser effect (NOE) were used to analyze the binding modes of two anti-oligosialic acid IgMs, 12E3 (group II) and A2B5 (group III) [51]. A2B5 favors a trisialic acid unit, while 12E3 favors ligands with more than five successive sialic-acid residues. A2B5 predominantly interacts with sialic-acid residues at the non-reducing end, while 12E3 binds to internal sialic-acid residues through C4–C8 moieties and *N*-acetyl groups. Thus, it is likely that A2B5 has exo-type and 12E3 endo-type interactions. scFv735 is similar to 12E3 in that it likely binds internal residues and directly interacts with OH4, OH7, OH8, a carboxyl group, and *N*-acetyl oxygen atoms. Notably, scFv735 interacts with all OH4 groups of the triplet, whereas 12E3 primarily interacts with *N*-acetyl groups of three sequential sialic-acid residues.

The functions of the polysialic acids are likely closely related to their three-dimensional structure; however, the conformations of polysialic acids remain a topic of debate [54–57]. Further analyses of the structure of polysialic acid-binding proteins in complex with their ligands and their dynamics will help to clarify the conformation of polysialic acid chains.

## 6. Additional Modifications

The interactions between linear polysaccharides and proteins can be regulated by additional modifications such as sulfation or branch formation of polysaccharide chains. Although the atomic details have been reported for certain heparin-binding proteins including antithrombin [58], FGF2 [59], and annexin [60], 3D structural information on the recognition mode of sulfated polysaccharides remains limited. The interaction with sulfated glycan is mostly electrostatic, attained by basic residues, Lys and Arg. However it is not so simple. Recent data suggest that FGF-2 binds not only heparin but also polysialic acid, possibly in different ways [61]. It is known that sulfation can modulate the conformation and dynamics of glycosaminoglycans, and such changes should closely correlate with different modes of binding to proteins. Deciphering how such a modification and the resulting changes in the dynamic properties regulate function is an important goal in the field of structural glycobiology.

## 7. Conclusions and Perspectives

Carbohydrate recognition by proteins is a fundamental biological phenomenon. Data is currently accumulating on the three-dimensional structure of polysaccharide-binding proteins, showing how the proteins bind to the carbohydrate ligands. X-ray crystallography and nuclear magnetic resonance (NMR) spectroscopy are the techniques of choice in determining protein oligosaccharide interactions. X-ray data provide a static view at the atomic level. In contrast, solution NMR provides information on structure and dynamics of small to medium-sized biological molecules. X-ray and solution NMR data are complementary, and a structure determined by one technique has greater import when supported by that of the other. X-ray data derives from a stable or one of the most stable conformations of the biomolecule, but it should be noted that crystal packing can induce an unstable conformation or an artificial interaction not observed in solution. However, there are very few studies to date on oligosaccharide–protein complexes analyzed by solution NMR.

It will be also important to determine the conformation of oligosaccharides in the absence of the cognate binding protein to ascertain the effect of binding. While NMR is suitable to obtain information on conformation in solution, multiple conformations can make analysis difficult and NMR parameters, such as NOE or scalar coupling constants, are typically insufficient to determine the structure. Experimental NMR analysis and molecular dynamics simulation together can provide a truer picture of the structure and dynamics of oligosaccharides in solution.
